# Age-dependent associations of peripheral immune-inflammatory indices with cerebrovascular remodeling after revascularization in ischemic moyamoya disease

**DOI:** 10.3389/fimmu.2026.1954649

**Published:** 2026-09-23

**Authors:** Wenjie Li, Xiaoliang Yin, Xin Chen, Huan Zhu, Qihang Zhang, Chenyu Zhu, Tao Wang, Yan Zhang, Jun Yang

**Affiliations:** 1 Department of Neurosurgery, Peking University Third Hospital, Beijing, China; 2 Department of Neurosurgery, Beijing Tiantan Hospital, Capital Medical University, Beijing, China; 3 Center for Precision Neurosurgery and Oncology of Peking University Health Science Center, Beijing, China

**Keywords:** arterial residual volume, collateralization angiogenesis ratio, Moyamoya disease, peripheral inflammatory indices, revascularization

## Abstract

**Objective:**

Peripheral immune-inflammatory activity may influence postoperative vascular remodeling in Moyamoya disease (MMD), but the relevant pathways may differ by developmental stage. We investigated age-dependent associations of blood cell-derived inflammatory indices with two complementary imaging outcomes: collateralization angiogenesis ratio (CAR) and arterial residual volume (ARV).

**Methods:**

This retrospective observational study included 108 patients with ischemic MMD who underwent revascularization (76 adults and 32 pediatric patients). Associations of neutrophil-to-lymphocyte ratio (NLR), platelet-to-lymphocyte ratio (PLR), lymphocyte-to-monocyte ratio (LMR), neutrophil-to-platelet ratio (NPR), and platelet-to-albumin ratio (PAR) with CAR and ARV were screened using Spearman correlations with Benjamini–Hochberg false-discovery-rate correction. Standardized multivariable linear models used HC3 robust standard errors and adjusted for age, sex, Suzuki stage, and follow-up. Effect modification was evaluated using cohort-by-marker and continuous age-by-marker interactions.

**Results:**

CAR and ARV did not differ between adults and pediatric patients (P = 0.583 and P = 0.853, respectively) and were uncorrelated within either cohort, supporting their interpretation as distinct remodeling dimensions. In unadjusted analyses, pediatric NPR correlated inversely with CAR (ρ=−0.431, P = 0.014), whereas adult PLR correlated inversely with ARV (ρ=−0.242, P = 0.035); neither association survived false-discovery-rate correction. After adjustment, pediatric NPR remained inversely associated with CAR (standardized β=−0.380, 95% CI −0.652 to −0.108; P = 0.006). The adult PLR–ARV association was borderline (β=−0.255, 95% CI −0.512 to 0.002; P = 0.052). PLR showed significant age-dependent effect modification: the PLR pediatric-cohort interaction was positive (β=0.547, 95% CI 0.112 to 0.981; P = 0.014), and the PLR continuous-age interaction was negative (β=−0.264, 95% CI −0.485 to −0.043; P = 0.019), indicating that the PLR–ARV relationship became more negative with increasing age.

**Conclusions:**

In this exploratory analysis, peripheral inflammatory indices were associated with different postoperative remodeling pathways according to developmental stage. The age-dependent PLR–ARV relationship was the most consistent interaction signal, whereas the adjusted pediatric NPR–CAR association suggests a potentially localized collateralization mechanism. However, these exploratory findings require validation in larger, prospectively characterized cohorts.

## 1 Introduction

Moyamoya disease (MMD) is a progressive steno-occlusive arteriopathy involving the terminal internal carotid arteries and their proximal branches, accompanied by the development of fragile basal collateral networks (1–3). In patients with ischemic presentation, current guidelines consider surgical revascularization reasonable for improving cerebral perfusion through direct flow augmentation, indirect collateral formation, or a combination of both (4–6). Pediatric evidence further indicates that direct, indirect, and combined procedures can all achieve effective revascularization, although angiographic and clinical responses remain heterogeneous (7). Some patients develop extensive extracranial-to-intracranial collaterals, whereas others retain or remodel native intracranial arteries to differing degrees (8). Understanding the biological factors associated with these divergent responses may refine postoperative assessment and generate patient-specific hypotheses for future therapeutic studies.

A single qualitative angiographic endpoint may not fully represent postoperative remodeling. Li et al. recently introduced a standardized digital subtraction angiography-based quantitative framework that evaluates the collateralization angiogenesis ratio (CAR) of the external carotid artery together with intracranial arterial residual volume (ARV) after revascularization (9, 10). In the present study, CAR was used to characterize newly developed extracranial-source collateral vasculature, whereas ARV represented residual or remodeled native intracranial arterial volume. These measures describe complementary aspects of vascular adaptation rather than interchangeable measures of the same process. Analyzing them in parallel offers a way to distinguish collateral angiogenesis from persistence or regression of the pre-existing arterial network.

Increasing evidence links moyamoya angiopathy to aberrant angiogenesis, endothelial dysfunction, and immune-inflammatory signaling (11–13). Peripheral blood cell-derived indices provide accessible summaries of systemic inflammatory and immune balance (14, 15). In a cross-sectional cohort of newly diagnosed MMD, platelet-to-lymphocyte ratio (PLR) and neutrophil-to-lymphocyte ratio (NLR) showed a nonlinear positive association, supporting the relevance of routinely available inflammatory indices in this population (16). Angiogenic factors may also relate to postoperative collateral formation; for example, peripheral soluble Tie-2 was associated with angiographic neovascularization after bypass surgery (17). Neutrophils, lymphocytes, monocytes, and platelets can influence endothelial activation, angiogenic signaling, thrombosis, and vascular remodeling (18–20). Their relative biological importance may vary with age because immune maturation, vascular plasticity, and the balance between direct and indirect revascularization differ substantially between pediatric and adult patients (9, 21, 22). Consequently, an association observed in one age group should not automatically be assumed to apply across the full age spectrum.

We therefore examined the relationships of five routinely derived peripheral inflammatory indices with CAR and ARV in a frozen cohort of adults and pediatric patients with ischemic MMD. The five indices (NLR, PLR, lymphocyte-to-monocyte ratio; LMR, neutrophil-to-platelet ratio; NPR, and platelet-to-albumin ratio; PAR) were selected *a priori* and are biologically motivated. Each captures a distinct balance between innate immune cells (neutrophils, monocytes), adaptive immune cells (lymphocytes), platelets, and albumin (an inflammation/nutrition-related carrier), and each is routinely available from a complete blood count plus albumin. The primary hypothesis was that age modifies the association between PLR and ARV, with a more negative relationship at older ages. A key secondary hypothesis was that NPR is inversely associated with CAR in pediatric patients. Broader correlation screening was considered exploratory and was interpreted together with multiplicity-adjusted results.

## 2 Methods

### 2.1 Study design and population

This retrospective observational study analyzed a two-center cohort of 134 consecutive patients with ischemic MMD who underwent surgical revascularization between January 2015 and May 2023. Ischemic MMD was an eligibility criterion for the entire study population; disease type was therefore not used as a subgrouping variable and no disease-type subgroup analysis was performed.

The diagnostic criteria for definite MMD followed the guidelines established by the Research Committee on MMD of Japan (23), and all cases were ultimately confirmed by digital subtraction angiography (DSA). Exclusion criteria were as follows: conditions that could significantly confound peripheral blood parameters, including acute infection, active autoimmune disease, malignancy, hematologic disorders, and recent use of medications (systemic corticosteroids; immunosuppressants; cytotoxic/chemotherapeutic agents; hematopoietic growth factors) known to have a substantial impact on blood cell counts.

This study was approved by the Institutional Review Board (IRB) of hospital, and all medical images and clinical data were fully anonymized. Digital Imaging and Communications in Medicine (DICOM) images were obtained from Picture Archiving and Communication System (PACS) servers in compliance with the Health Insurance Portability and Accountability Act.

### 2.2 Clinical, surgical, and laboratory variables

Baseline variables included age, sex, Matsushima grade, Suzuki stage, follow-up duration, surgical side, and surgical method. Age group was coded as adult or pediatric. Suzuki stage described angiographic disease severity. To ensure accurate staging and grading, two experienced neuroradiologists (each with >10 years of experience) independently reviewed the DSA images to determine the Suzuki stage and Matsushima grade. Any discrepancies were resolved by consensus with a third senior radiologist.

All enrolled patients underwent indirect cerebral revascularization surgery, specifically encephaloduroarteriosynangiosis (EDAS). The only revascularization technique used in this cohort. All procedures were unilateral and were performed at a single center by a single standardized surgical team using a consistent protocol, with no variation in operative technique, donor vessel selection, or the number of treated territories.The EDAS procedure was performed by attaching the superficial temporal artery to the cortical surface of the brain, thereby promoting the gradual angiogenesis of new collateral vessels and improving cerebral blood flow over time.

For all eligible participants, fasting venous blood samples were collected within one week before the index surgery. To avoid potential confounding from acute illness or recent intervention, individuals with any major clinical event or laboratory abnormality in the 30 days preceding surgery were excluded from the study. Hematological parameters, including total leukocyte counts, neutrophil counts, lymphocyte counts, monocyte counts, platelet counts, and serum albumin levels, were measured using an auto-analyzer (XE-2100, Sysmex, Kobe, Japan) in the central laboratory. From these measurements, the following composite ratios were derived: NLR, PLR, lymphocyte-to-monocyte ratio (LMR), NPR, and platelet-to-albumin ratio (PAR). All ratios were treated as continuous variables in the analyses. Because serum albumin data were not available for a subset of patients, PAR calculations relied on complete cases; therefore, any analysis involving PAR was restricted to participants with available albumin measurements.

### 2.3 Postoperative vascular remodeling outcomes

Postoperative DSA was performed at each patient’s routine clinical follow-up, and the recorded follow-up time corresponds to the time at which the follow-up angiography was obtained (median 10.0 months [IQR 7.0–14.0] in adults; 8.0 months [IQR 5.0–13.25] in pediatric patients). Two quantitative postoperative imaging outcomes were evaluated using the digital subtraction angiography (DSA)-based framework described by Li et al. (9). The CAR represented the relative extent of collateral vasculature attributed to postoperative angiogenesis from external carotid artery sources, while the ARV represented the residual or remodeled volume of native intracranial arteries. CAR and ARV were treated as parallel, biologically complementary outcomes throughout the analysis. Angiographic data were reviewed for eligibility; patients lacking separate internal and external carotid artery injections or with incomplete angiographic cycles were excluded. Each dynamic DSA run was first converted into 1024 × 1024 pixels JPEG images at a 1:1 ratio. To focus on the revascularization territory, only the region above the skull base origin was retained. Quality control was performed by a clinician with five years of experience, and images containing venous or sinus phases were discarded. Background normalization was achieved by subtracting the initial mask image from all subsequent frames, producing a uniform white background with black vessels, and pixel intensities were scaled to a 0–255 range. A fixed pixel threshold of 220 was then applied to segment the vasculature, and the number of pixels above this threshold was counted separately on anterior-posterior and lateral projections. Head size was standardized using the Segment Anything Model to delineate the skull contour; the head pixel count (Hp) was used to normalize vascular measurements. The pixel counts of the external carotid artery-derived vessels (collateralization) on lateral (LEp) and anterior (AEp) views were averaged to derive CAR, while the pixel counts of the intracranial arteries on lateral (LIp) and anterior (AIp) views were averaged to derive ARV, according to the formulas: CAR = (LEp + AEp)/(2 × Hp) × 100%; ARV = (LIp + AIp)/(2 × Hp) × 100%. All image processing was performed using Python 3.8.12 on a workstation equipped with AMD Ryzen™ 9 7950X CPU and an NVIDIA GeForce RTX 4090 GPU.

### 2.4 Statistical analysis

Categorical variables were summarized as counts and percentages, and continuous variables were summarized as medians with interquartile ranges (IQRs). Between-cohort comparisons of categorical variables were performed using Pearson’s chi-square test or Fisher’s exact test, as appropriate. Continuous variables were compared using the Mann–Whitney U test. Spearman rank-correlation coefficients (rho) were used to assess the associations between CAR and ARV in the adult cohort, pediatric cohort, and full study population. Cohort-specific multivariable linear regression models were used to evaluate the focused associations. Heteroscedasticity-consistent HC3 standard errors were used to calculate 95% confidence intervals and P values. Regression results are reported as standardized beta coefficients with robust standard errors, 95% confidence intervals, and adjusted R² values. All tests were two-sided, and P < 0.05 was considered statistically significant. The analyses were conducted using IBM SPSS Statistics, version 26.0 (IBM Corp., Armonk, NY, USA).

## 3 Results

### 3.1 Cohort characteristics

The study was conducted at a single center, and all consecutive patients meeting the eligibility criteria during this period were enrolled. A total of 134 patients were screened; 11 were excluded for missing blood indicators, 9 for incomplete DSA sequences, and 6 for head-mask generation failure, yielding the 108 included patients. The study included 76 adults and 32 pediatric patients (**Table 1**). Sex distribution was similar between cohorts (male: 47.4% vs 50.0%; P = 0.803). Median age was 43.5 years (interquartile range [IQR], 34.0–49.0) in adults and 10.5 years (IQR, 7.0–12.25) in pediatric patients (P<0.001). Matsushima grade (P = 0.261), Suzuki stage (P = 0.326), follow-up duration (10 [IQR, 7–14] vs 8 [IQR, 5–13.25] months; P = 0.141), and surgical side (P = 0.973) did not differ significantly.

**Table 1:** Baseline characteristics of the adult and pediatric MMD cohorts.

Characteristic	Adult (n = 76)	Pediatric (n = 32)	P value
Sex, n (%)			0.803
Male	36 (47.4)	16 (50.0)	
Female	40 (52.6)	16 (50.0)	
Age, years	43.5 [34.0, 49.0]	10.5 [7.0, 12.25]	<0.001
Matsushima classification, n (%)			0.261
Grade B	12 (15.8)	8 (25.0)	
Grade A	64 (84.2)	24 (75.0)	
Suzuki staging, n (%)			0.326
Stage III	7 (9.2)	0 (0.0)	
Stage IV	53 (69.7)	23 (71.9)	
Stage V	14 (18.4)	8 (25.0)	
Stage VI	2 (2.6)	1 (3.1)	
Follow-up time, months	10.0 [7.0, 14.0]	8.0 [5.0, 13.25]	0.141
Surgical side, n (%)			0.973
Left	52 (68.4)	22 (68.8)	
Right	24 (31.6)	10 (31.2)	

MMD, Moyamoya disease; n, number of patients. the surgical side (left/right) refers to unilateral left-sided vs right-sided procedures.

Adults had higher NLR (1.973 [IQR, 1.467–2.547] vs 1.360 [IQR, 1.010–2.518]; P = 0.047) and NPR (.016 [IQR,.012–.019] vs.011 [IQR,.009–.016]; P = 0.001), whereas pediatric patients had higher PAR (6.328 [IQR, 5.629–7.441] vs 5.227 [IQR, 4.596–6.511]; P = 0.007). PLR (P = 0.739) and LMR (P = 0.065) did not differ significantly.

### 3.2 Distribution and independence of CAR and ARV

Median ARV was 0.039 (IQR, 0.026–0.058) in adults and 0.040 (IQR, 0.029–0.053) in pediatric patients (P = 0.853). Median CAR was 0.060 (IQR, 0.035–0.104) and 0.073 (IQR, 0.034–0.112), respectively (P = 0.583) (**Table 2**, **Figure 1**). Thus, neither remodeling outcome showed a detectable difference in overall level between age cohorts.

**Table 2:** Comparison of vascular remodeling parameters between adult and pediatric MMD cohorts.

Parameter	Adult (n = 76)	Pediatric (n = 32)	P value
ARV	0.039 [0.026, 0.058]	0.040 [0.029, 0.053]	0.853
CAR	0.060 [0.035, 0.104]	0.073 [0.034, 0.112]	0.583

MMD, Moyamoya disease; n, number of patients; ARV, arterial residual volume; CAR, collateralization angiogenesis ratio.

**Figure 1:**
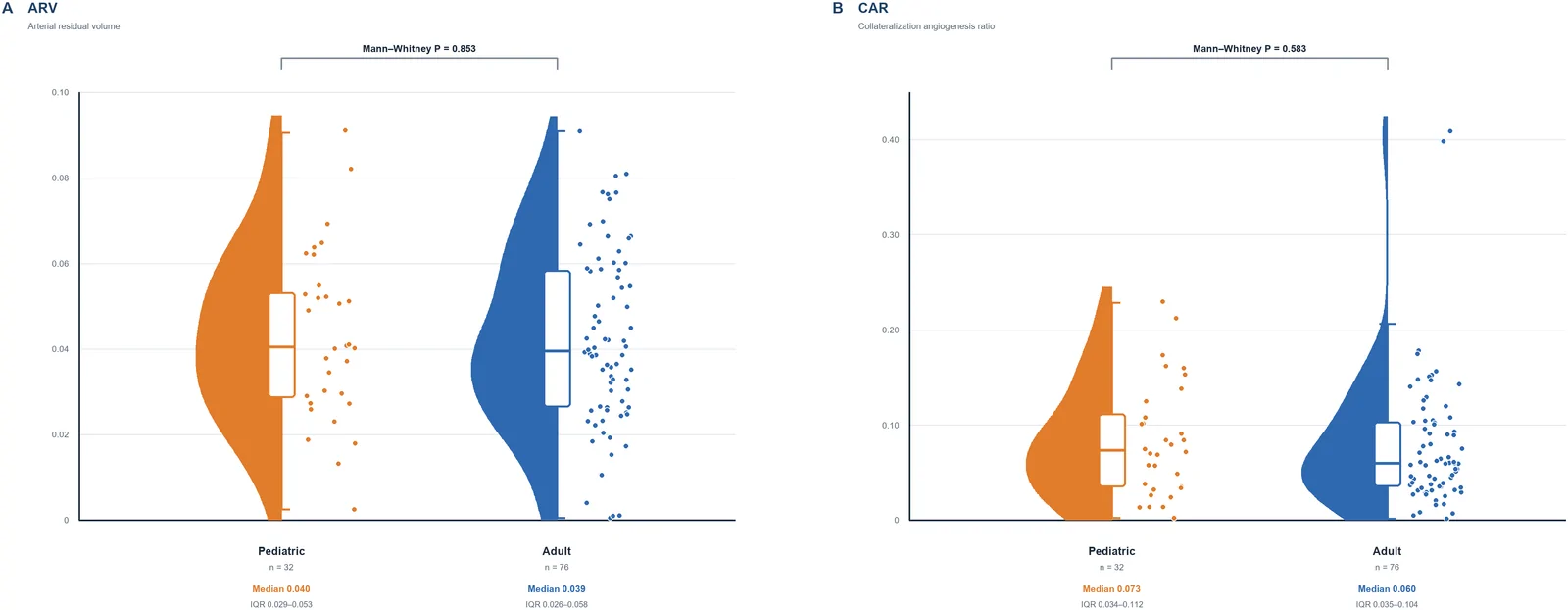
Raincloud plots compare pediatric patients (orange; n = 32) and adults (blue; n = 76) for **(A)** ARV and **(B)** CAR. Each plot combines a half-violin density distribution, a box plot, and jittered individual observations; the box denotes the interquartile range and the internal line denotes the median. Median ARV was 0.040 (interquartile range, 0.029–0.053) in the pediatric cohort and 0.039 (0.026–0.058) in adults (Mann–Whitney U test, P = 0.853). Median CAR was 0.073 (0.034–0.112) in the pediatric cohort and 0.060 (0.035–0.104) in adults (P = 0.583).

CAR and ARV were essentially uncorrelated in adults (ρ=−0.037, P = 0.754), pediatric patients (ρ=0.018, P = 0.924), and the full cohort (ρ=−0.015, P = 0.880). This empirical independence supports their use as distinct, complementary dimensions of postoperative remodeling rather than as redundant measures.

### 3.3 Cohort-specific correlations with peripheral inflammatory indices

In pediatric patients, NPR was inversely correlated with CAR (ρ=−0.431, P = 0.014), whereas its association with ARV was weak (ρ=−0.108, P = 0.557). No other pediatric correlation reached nominal significance. The largest additional pediatric estimates were PLR with ARV (ρ=0.328, P = 0.066) and PAR with CAR (ρ=0.329, P = 0.146).

In adults, PLR was inversely correlated with ARV (ρ=−0.242, P = 0.035), whereas its association with CAR was negligible (ρ=0.017, P = 0.884). Other adult correlations were not statistically significant. The pediatric NPR–CAR and adult PLR–ARV associations did not survive Benjamini–Hochberg correction (q=0.137 and q=0.350, respectively); no screened correlation remained significant after FDR adjustment (**Table 3**, **Figure 2**).

**Table 3:** Correlations between peripheral immune-inflammatory markers and vascular remodeling parameters.

Marker	Pediatric ARV rho (P value)	Adult ARV rho (P value)	Pediatric CAR rho (P value)	Adult CAR rho (P value)
NLR	0.114 (0.536)	-0.171 (0.140)	-0.275 (0.128)	-0.026 (0.822)
PLR	0.328 (0.066)	-0.242 (0.035)	0.069 (0.709)	0.017 (0.884)
LMR	-0.245 (0.176)	0.016 (0.889)	0.254 (0.161)	0.132 (0.255)
NPR	-0.108 (0.557)	0.066 (0.572)	-0.431 (0.014)	-0.059 (0.614)
PAR	0.258 (0.258)	-0.079 (0.548)	0.329 (0.146)	0.166 (0.204)

ARV, arterial residual volume; CAR, collateralization angiogenesis ratio; NLR, neutrophil-to-lymphocyte ratio; PLR, platelet-to-lymphocyte ratio; LMR, lymphocyte-to-monocyte ratio; NPR, neutrophil-to-platelet ratio; PAR, platelet-to-albumin ratio.

**Figure 2:**
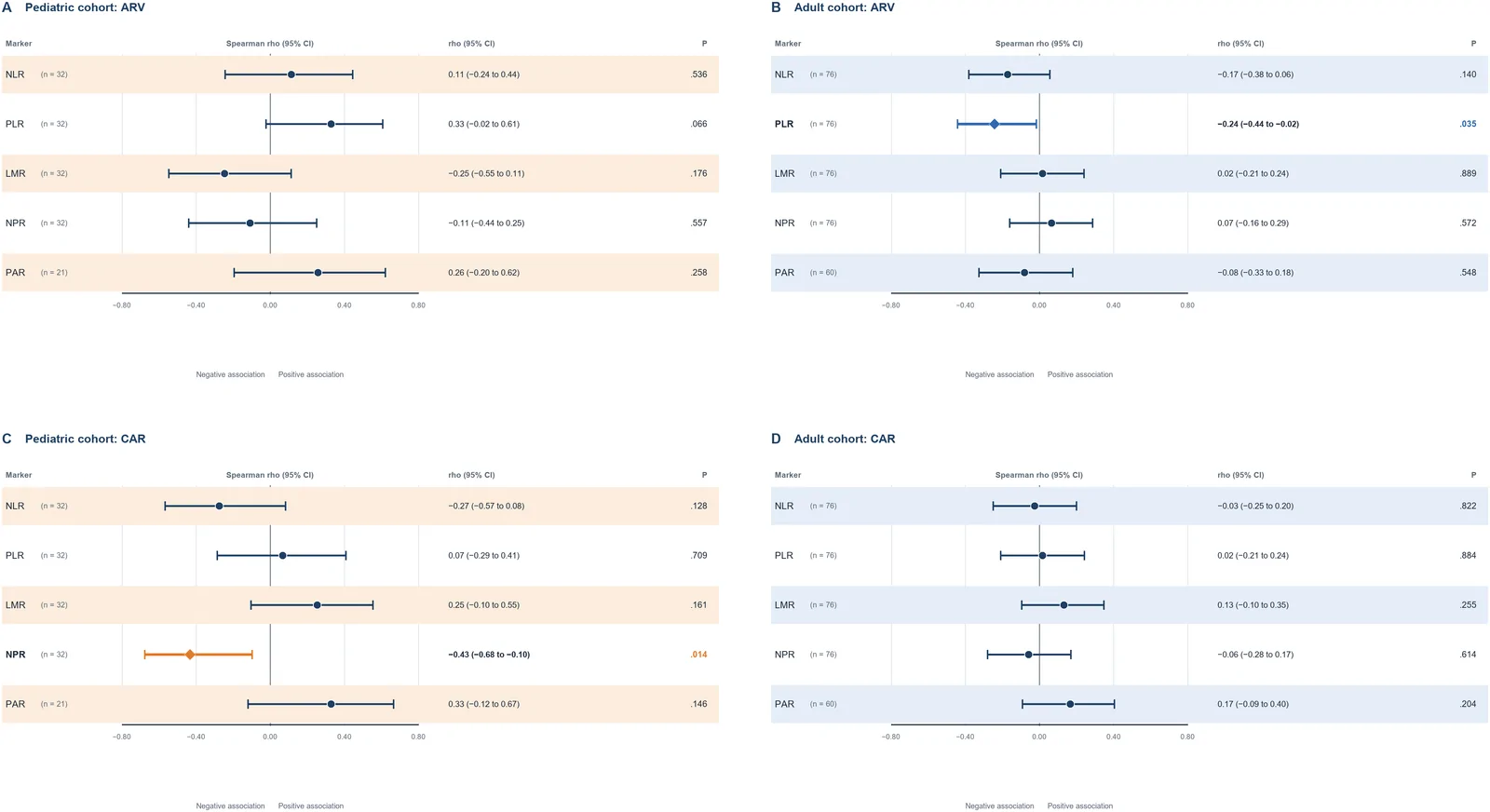
Forest plots show unadjusted Spearman rank-correlation coefficients (rho) and 95% confidence intervals for NLR, PLR, LMR, NPR, and PAR in relation to **(A)** ARV in pediatric patients, **(B)** ARV in adults, **(C)** CAR in pediatric patients, and **(D)** CAR in adults. Circles indicate correlation estimates, horizontal bars indicate 95% confidence intervals, and the vertical reference line denotes rho = 0. Diamonds and colored text identify nominal associations at P < 0.05. The principal nominal findings were an inverse PLR–ARV correlation in adults (rho = −0.24, 95% CI −0.44 to −0.02; P = 0.035) and an inverse NPR–CAR correlation in pediatric patients (rho = −0.43, 95% CI −0.68 to −0.10; P = 0.014). PAR analyses were based on available cases (pediatric n = 21; adult n = 60); all other analyses included 32 pediatric patients and 76 adults. No correlation remained statistically significant after Benjamini–Hochberg false-discovery-rate correction.

### 3.4 Adjusted cohort-specific associations

In the pediatric CAR model (n=32), higher NPR remained independently associated with lower CAR after adjustment for age, sex, Suzuki stage, and follow-up (standardized β=−0.380, robust standard error [SE]=0.139, 95% CI −0.652 to −0.108; P = 0.006). The model had limited overall explanatory capacity (adjusted R²=0.024).

In the adult ARV model (n=76), higher PLR was associated with lower ARV at borderline statistical significance (standardized β=−0.255, robust SE = 0.131, 95% CI −0.512 to 0.002; P = 0.052; adjusted R²=0.017). Other covariates in both models were not statistically significant (**Table 4**, **Figure 3**).

**Table 4:** Multivariable linear regression analyses of pediatric MMD CAR and adult MMD ARV.

Model	Predictor	Standardized beta	Robust SE	95% CI	P value	Adjusted R^2^
Pediatric CAR (n = 32)	NPR	-0.380	0.139	-0.652 to -0.108	0.006	0.024
	Age	-0.089	0.191	-0.464 to 0.286	0.642	
	Female sex	0.113	0.445	-0.758 to 0.985	0.799	
	Suzuki stage	-0.034	0.294	-0.609 to 0.542	0.909	
	Follow-up time	0.221	0.319	-0.405 to 0.847	0.489	
Adult ARV (n = 76)	PLR	-0.255	0.131	-0.512 to 0.002	0.052	0.017
	Age	0.167	0.113	-0.055 to 0.388	0.140	
	Female sex	0.051	0.283	-0.504 to 0.606	0.856	
	Suzuki stage	0.010	0.111	-0.208 to 0.228	0.928	
	Follow-up time	0.033	0.143	-0.247 to 0.313	0.819	

MMD, Moyamoya disease; n, number of patients; CAR, collateralization angiogenesis ratio; ARV, arterial residual volume; NPR, neutrophil-to-platelet ratio; PLR, platelet-to-lymphocyte ratio; CI, confidence interval; SE, standard error.

**Figure 3:**
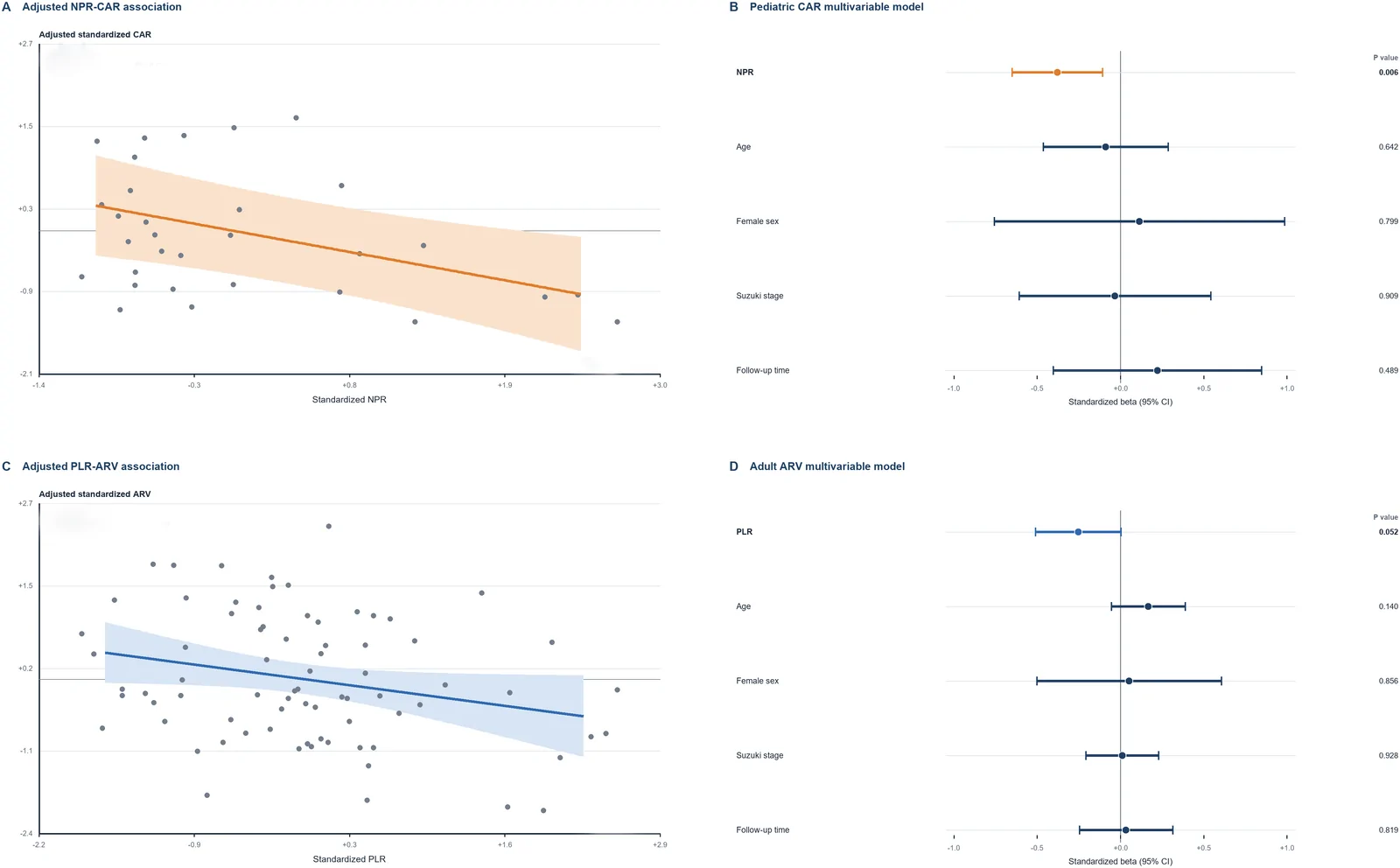
**(A)** Added-variable plot showing the adjusted association between standardized NPR and standardized CAR in the pediatric cohort (n = 32). Points represent covariate-adjusted partial residuals, the orange line represents the fitted association, and the shaded band represents its 95% confidence interval. Higher NPR was independently associated with lower CAR (standardized beta = −0.380, 95% CI −0.652 to −0.108; P = 0.006). **(B)** Forest plot of the pediatric CAR multivariable model; points are standardized regression coefficients and bars are 95% confidence intervals. The model adjusted for age, sex, Suzuki stage, and follow-up duration and had an adjusted R² of 0.024. **(C)** Added-variable plot for standardized PLR and standardized ARV in adults (n = 76), showing a borderline inverse association (standardized beta = −0.255, 95% CI −0.512 to 0.002; P = 0.052). **(D)** Forest plot of the corresponding adult ARV multivariable model, adjusted for the same covariates, with an adjusted R² of 0.017. The vertical line in the forest plots denotes a standardized beta of 0.

### 3.5 Age-dependent effect modification

The adjusted PLR–ARV interaction was significant under both age parameterizations (**Table 5**, **Figure 4**). Relative to adults, the pediatric PLR slope was 0.547 standard-deviation units higher (PLR pediatric cohort: β=0.547, robust SE = 0.222, 95% CI 0.112 to 0.981; P = 0.014; adjusted R²=0.042). In the continuous-age model, the interaction was negative (PLR age: β=−0.264, robust SE = 0.113, 95% CI −0.485 to −0.043; P = 0.019; adjusted R²=0.033). These coefficients are directionally consistent: the PLR–ARV relationship was less negative or more positive in pediatric patients and became progressively more negative as age increased.

**Table 5:** Adjusted interaction analyses of PLR-ARV and NPR-CAR associations.

Outcome	Marker	Interaction term	n	Standardized beta	Robust SE	95% CI	P value	Adjusted R^2^
ARV	PLR	PLR x pediatric cohort	108	0.547	0.222	0.112 to 0.981	0.014	0.042
ARV	PLR	PLR x continuous age	108	-0.264	0.113	-0.485 to -0.043	0.019	0.033
CAR	NPR	NPR x pediatric cohort	108	-0.382	0.201	-0.775 to 0.011	0.057	-0.003
CAR	NPR	NPR x continuous age	108	0.207	0.106	-0.001 to 0.415	0.052	0.011

ARV, arterial residual volume; CAR, collateralization angiogenesis ratio; PLR, platelet-to-lymphocyte ratio; NPR, neutrophil-to-platelet ratio; CI, confidence interval; SE, standard error; n, number of patients.

**Figure 4:**
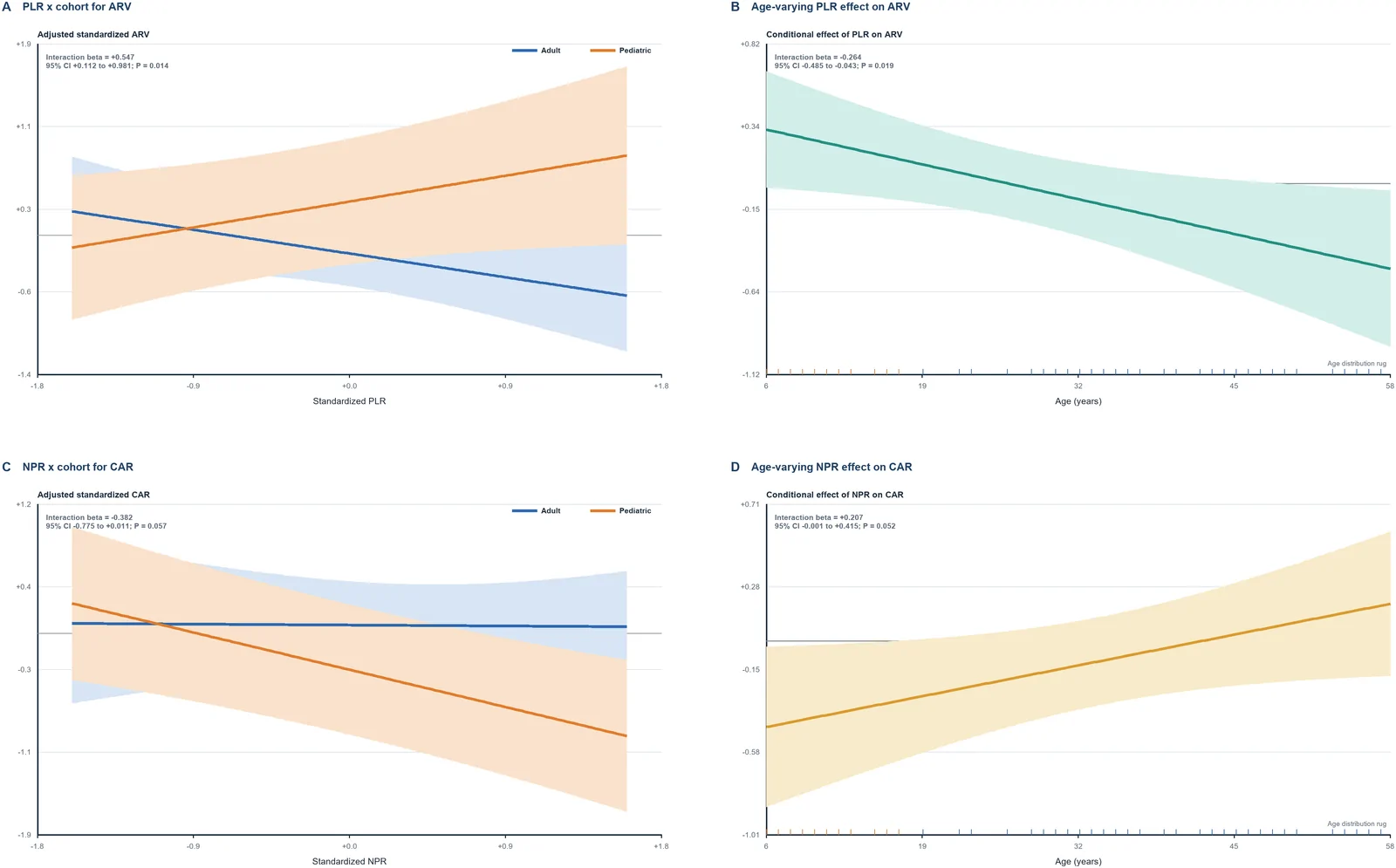
**(A)** Cohort-specific adjusted standardized ARV across standardized PLR. Blue denotes adults and orange denotes pediatric patients; solid lines are adjusted predictions and shaded bands are 95% confidence intervals. The PLR-by-pediatric-cohort interaction was significant (interaction beta = 0.547, 95% CI 0.112–0.981; P = 0.014), indicating that the PLR–ARV slope was higher in pediatric patients than in adults. **(B)** Conditional effect of PLR on ARV across continuous age. The negative PLR-by-age interaction (beta = −0.264, 95% CI −0.485 to −0.043; P = 0.019) indicates that the PLR–ARV association becomes progressively more negative with increasing age. **(C)** Cohort-specific adjusted standardized CAR across standardized NPR. The NPR-by-pediatric-cohort interaction suggested a more negative NPR–CAR slope in pediatric patients but did not meet the prespecified significance threshold (beta = −0.382, 95% CI −0.775 to 0.011; P = 0.057). **(D)** Conditional effect of NPR on CAR across continuous age. The NPR-by-age interaction was similarly borderline (beta = 0.207, 95% CI −0.001 to 0.415; P = 0.052), suggesting attenuation of the negative NPR–CAR association with increasing age. Rug marks in panels B and D show the observed age distribution (orange, pediatric; blue, adult), and the horizontal line denotes a conditional effect of 0. Interaction models were adjusted for age, sex, Suzuki stage, and follow-up duration, with HC3 heteroscedasticity-robust standard errors; adults were the reference group in cohort-interaction models.

For CAR, the NPR pediatric-cohort interaction was β=−0.382 (robust SE = 0.201, 95% CI −0.775 to 0.011; P = 0.057; adjusted R²=−0.003), and the NPR age interaction was β=0.207 (robust SE = 0.106, 95% CI −0.001 to 0.415; P = 0.052; adjusted R²=0.011). These near-significant, parameterization-dependent estimates did not meet the prespecified 0.05 threshold and therefore provide insufficient evidence for a general age interaction. They are more consistent with a localized pediatric NPR–CAR signal that requires replication.

## 4 Discussion

This study identified developmentally distinct associations between peripheral inflammatory indices and postoperative cerebrovascular remodelling in patients with ischemic MMD. Four principal findings emerged. First, CAR and ARV were uncorrelated and did not differ in overall magnitude between adult and pediatric patients, supporting their interpretation as complementary rather than redundant outcomes. Second, NPR was inversely associated with CAR in pediatric patients, and this association persisted after multivariable adjustment. Third, the association between PLR and ARV in adults was negative but of borderline significance after adjustment. Fourth, and notably, interaction analyses consistently demonstrated that the PLR–ARV association became more negative with increasing age.

The separation of CAR and ARV is of conceptual importance and extends the quantitative vascular framework introduced by Li et al. (9, 10). CAR is designed to capture collateral angiogenesis originating from extracranial sources, whereas ARV reflects persistence or remodelling of the native intracranial arterial tree. A patient may develop abundant surgical collaterals without a proportional change in native arterial volume, or may retain native vessels despite limited collateral proliferation (24, 25). The near-zero correlations between CAR and ARV in both age groups support this dual-axis framework. Future studies that collapse postoperative remodelling into a single composite endpoint may obscure pathway-specific biological associations.

The adjusted inverse NPR–CAR association in pediatric patients suggests that the balance between neutrophils and platelets may be linked to collateral angiogenesis during childhood. A lower NPR may reflect a platelet-dominant milieu, a reduced neutrophil burden, or both. Platelets participate in endothelial signalling and release pro-angiogenic mediators, whereas excessive neutrophil-driven inflammation can contribute to endothelial injury and dysregulated vascular repair (13, 26, 27). These mechanisms render the observed direction biologically plausible, although the ratio cannot distinguish which cellular component is causal. This mechanism is hypothesis-generating: NPR is a ratio and therefore cannot, on its own, distinguish a platelet-dominant milieu from a reduced neutrophil burden. Platelets release pro-angiogenic mediators and support endothelial signalling, whereas excessive neutrophil-driven inflammation can contribute to endothelial injury and dysregulated repair; thus a lower NPR is directionally consistent with a relatively greater platelet influence, but this remains inferential and cannot be resolved by a ratio. Moreover, the nominal correlation did not survive FDR correction, the pediatric sample was small, and the model accounted for a limited proportion of variance. This finding should therefore be regarded as a focused hypothesis for validation rather than as evidence of a clinically actionable biomarker.

The PLR–ARV interaction provided a clearer age-dependent signal. The positive PLR-by-pediatric-cohort coefficient indicates that the pediatric PLR slope was higher—less negative or more positive—than the adult slope. The negative PLR-by-continuous-age coefficient expresses the same pattern on a continuous scale: for each standardized increment in age, the association between PLR and ARV shifted in a more negative direction. Thus, the two interaction formulations are concordant. Together, they suggest that a higher PLR is increasingly associated with a lower residual native arterial volume as patients become older. Age-related changes in immune balance, endothelial resilience, arterial plasticity, and the relative contribution of direct versus indirect revascularization are potential explanations; the available data cannot differentiate among these mechanisms.

The NPR–CAR interactions require a different interpretation. Although the pediatric cohort-specific association was significant after adjustment, the age-interaction terms did not attain the conventional significance threshold and were not uniformly directional in a manner supporting a simple monotonic age effect. This pattern suggests that the NPR–CAR relationship may reflect a mechanism concentrated within the pediatric cohort rather than a smooth gradient across the entire age span. A threshold phenomenon, nonlinear developmental change, sampling variation, or residual confounding could each produce such a pattern. Expansion of the pediatric sample and flexible nonlinear age modeling are necessary before age modification can be confirmed.

Postoperative cerebrovascular remodeling is likely governed by vascular anatomy, hemodynamics, operative technique, genetic susceptibility, growth factors, and local tissue responses in addition to systemic blood indices (28–31). The present inflammatory markers capture only a small fraction of this complexity. The significant adjusted association observed in the pediatric cohort and the concordant PLR interaction models justify further investigation but do not support stand-alone prediction or clinical decision-making.

This study has several strengths. The analysis was restricted to ischemic MMD, thereby avoiding etiological subgroup comparisons beyond the scope of the research question. It examined two biologically distinct remodeling outcomes, employed prespecified covariate adjustment, reported standardized effect sizes, used HC3 robust standard errors, applied multiplicity correction for the broad correlation screen, and implemented both categorical and continuous formulations of age interaction. The consistency of the PLR–ARV interaction across both formulations strengthens the internal coherence of that result. Several limitations should be acknowledged. The retrospective, single-dataset design precludes causal inference and external validation. The adjusted R^2^ values were low, that the effect estimates are not clinically interpretable effect sizes, and that the associations do not support clinical prediction. Exploratory screening was FDR-corrected, whereas focused hypotheses (adjusted CAR–ARV association and its interaction with developmental stage) were tested without multiplicity correction, rendering them exploratory and hypothesis-generating and requiring validation in independent cohorts.

Future work should prospectively enroll larger pediatric and adult cohorts, standardize the timing of blood sampling and postoperative imaging, quantify imaging reproducibility, and incorporate hemodynamic and anatomic variables. The primary confirmatory target should be the age-dependent PLR–ARV interaction, with the pediatric NPR–CAR association serving as a key secondary hypothesis.

## 5 Conclusions

CAR and ARV represent distinct dimensions of postoperative vascular remodeling in ischemic MMD. The association between PLR and ARV became more negative with increasing age, providing the most consistent evidence of developmental effect modification. NPR was independently and inversely associated with CAR in pediatric patients, but the corresponding interaction results were borderline and require larger pediatric samples. These findings support age-aware, pathway-specific investigation of immune-inflammatory contributions to remodeling after revascularization, while remaining exploratory until prospectively replicated.

## Data Availability

The raw data supporting the conclusions of this article will be made available by the authors, without undue reservation.
